# The Conceit of a Microbe—A Fable

**Published:** 1901-02

**Authors:** 


### The Conceit of a Microbe—A Fable.

"Wa'd some power the giftie gie us,
To see oursels as ithers see us.
'Twould fra' mony a blunder free us, and foolish notion."
—*Bobby B.*

"What a mighty fish a frog is."—*Josh Billings.*

**THE MAN WHO KNEW IT ALL.**

ONCE UPON A TIME there lived Out West, A Man Who Knew It All. He was a Doctor, of course; half homeopath, half hypnotist and half Christian Scientist, whatever that may be. He had a cinch on Wisdom and a corner on Knowledge. No one else on the Face of the Earth knew Anything Whatever; no, not even a little Bit. There was a universal Knowledge Famine. He was Sorry for them, and was Lonesome, but he couldn't help it,—he was Born So. "The world is asleep," he said, and he was going to "Wake them Up." He was going to Knock some Knowledge, some Common Sense, he said, into their heads, and he did it with A Stuffed Club. He called it "A Stuffed Club for Everybody." He was impartial. He made no exceptions. Everybody must have Some. He administered it like the Spaniards did Chistianity to the Mexicans,—with a Club.

The Stuffed Club For Everybody was a little 4x6 pamphlet of some 32 pages, issued monthly. We saw the "Stuff,"—it was all stuff,—but the club looked more like a Feather. I used to get three copies every month. I felt inclined to get Huffy; I felt that it was a Reflection on me. It indicated that the Man Who Knew It All thought me a particularly Tough Case. It implied that I did not know Anything Whatever, a great deal worse than the other fellows didn't know it. But being an Editor, I took my medicine like a man, for I want to Know, you know, and am Willing to Learn; even to have Knowledge knocked into my head. I would prefer that way to the Way the fellow had to take his Soup,—don't you know.

Some of the Great Truths which the Man who Knew It All clubbed into the heads of all the world with his Stuffed Club were:

Medicine is a Mistake. There is no such Thing as Disease,—it's only Malnutrition; that's All. Microbes are a Myth. Pathogenic bacteria are a Boog-a Boo. Cancer of the Rectum can be cured by Diet Alone,—with a big D and a big A; HE does it. Vaccination is All Wrong. It kills People and doesn't prevent Small-Pox. Jenner was a Fool. Pasteur was an Idiot. Lister was a Lunatic. Newton, Lock and Bacon were Mistaken. Spencer and Darwin and Huxley and Haeckel and Wallace were,—oh well, HE had Investigated Evolution, and, (with a wave of the hand,) "there is nothing in it, n-o-t-h-i-n-g In it." Copernicus, Galileo, Herschel, Leverrier, Kant, Laplace, Simon Newcomb and Professor Barnard,—a-l-l Wrong. "The Sun Do move," said Brother Jasper, and so thought this Jasper-with-a-club. Like the country Preacher who, preaching from Paul's Epistles to The Marines,—he begged to differ with Brother Paul.

"The World's Asleep," he said, and HE was going to "Wake them Up,"—with his Stuffed Club.

A Little Cock cricket stood at the Door of his little Hole-in-the-ground, and cheerfully chirped his little chirp with great Gusto. Retiring then to the Bosom of his Family, he remarked with a satisfied Smile to Mrs. Cricket, who was Deep in Mrs. Eddy's Book: "I guess, my dear, that THAT blast I just blasted will shake the Pillars of the Universe and wake the World."

Who has not read Martin Chuzzlewit? Martin and Mark Tapley, on their way to Eden, (which I always believed was in Texas,) were accosted on the cars by the natives, for whom Mr. Lafayette Kettle and General Choke were spokesmen. Learning by inquiry that Martin and Mark were Englishmen, Mr. Lafayette Kettle said:

"How's Queen Victoria"?

"In good health, I believe," said Martin.

"Queen Victoria won't shake in her royal shoes at all, when she hears tomorrow named," observed the stranger,—"No."

"Not that I'm aware of; why should she"?

"She won't be taken with a cold chill, when she realizes what's being done in these diggin's," said the stranger,—"No."

"No," said Martin, "I think I could take my oath on that."

The strange gentleman looked at him as if in pity for his ignorance or prejudice, and said:

"Well, sir, I tell you this: There aint a en-gine, with its biler bust, in God A'Mighty's free U-nited States, so fixed, and nipped, and frizzled to a most e-ternal smash, as that young critter, in her luxurious lo-cation in the Tower of London, will be, when she reads the next double-extra Watertoast Gazette." [The Watertoast Gazette was a primitive Stuffed Club, I suppose.—Editor.]

"Upon my word," said Martin, "I have only to say that I never heard of Queen Victoria reading the Whats-his-name-Gazette, and I should scarcely think it probable."

General Choke smiled upon the rest of the party, and said, in patient and benign explanation: "It's *sent* to her, sir. It's *sent* to her. Per mail."

The-Man-With-A-Stuffed-Club who Knew-it-All,—died of Wisdom. That is,—he was too Wise to be vaccinated, and he died of small-pox; same thing.

